# Redox-Specialized Bacterioplankton Metacommunity in a Temperate Estuary

**DOI:** 10.1371/journal.pone.0122304

**Published:** 2015-04-10

**Authors:** Peeter Laas, Jaak Simm, Inga Lips, Urmas Lips, Veljo Kisand, Madis Metsis

**Affiliations:** 1 Marine Systems Institute at Tallinn University of Technology, Tallinn, Estonia; 2 Department of Electrical Engineering (ESAT), STADIUS Center for Dynamical Systems, Signal Processing, and Data Analytics, KU Leuven, Leuven, Belgium; 3 iMinds Medical IT, Leuven, Belgium; 4 Department of Gene Technology, Tallinn University of Technology, Tallinn, Estonia; 5 Institute of Technology at University of Tartu, Tartu, Estonia; 6 Institute of Mathematics and Natural Sciences, Tallinn University, Tallinn, Estonia; American University in Cairo, EGYPT

## Abstract

This study explored the spatiotemporal dynamics of the bacterioplankton community composition in the Gulf of Finland (easternmost sub-basin of the Baltic Sea) based on phylogenetic analysis of 16S rDNA sequences acquired from community samples via pyrosequencing. Investigations of bacterioplankton in hydrographically complex systems provide good insight into the strategies by which microbes deal with spatiotemporal hydrographic gradients, as demonstrated by our research. Many ribotypes were closely affiliated with sequences isolated from environments with similar steep physiochemical gradients and/or seasonal changes, including seasonally anoxic estuaries. Hence, one of the main conclusions of this study is that marine ecosystems where oxygen and salinity gradients co-occur can be considered a habitat for a cosmopolitan metacommunity consisting of specialized groups occupying niches universal to such environments throughout the world. These niches revolve around functional capabilities to utilize different electron receptors and donors (including trace metal and single carbon compounds). On the other hand, temporal shifts in the bacterioplankton community composition at the surface layer were mainly connected to the seasonal succession of phytoplankton and the inflow of freshwater species. We also conclude that many relatively abundant populations are indigenous and well-established in the area.

## Introduction

The world’s oceans are the cradle of life; hence, the evolution of aquatic microorganisms for 3.5 billion years has produced enormous diversity and functional plasticity, only recently assessed by the sequencing of metagenomic DNA (pioneered by [1]). There are many aspects of microbial life that make the ecology of microorganisms different from that of macroorganisms [2], including intercontinental dispersion by winds [3] and the capability to persist in environmentally hostile conditions over a long period of time [4]. Aquatic microbes are essential for life on Earth [5,6] and therefore unveiling the mechanisms underlying the spatiotemporal dynamics of bacterioplankton community composition (BCC) remains the one of the most important issues in aquatic microbial ecology.

Over the last few decades, advances in sequencing technologies have revolutionized the power of the identification process for microorganisms and thereby revealed tremendous microbial diversity and plasticity in aquatic environments [7]. 16S rRNA gene-based investigations have contributed a massive number of sequences to databases and have revealed a comprehensive uncultured microbial diversity [8,9]. The ease of microbial community profiling has been effectively utilized to determine the biogeographic patterns of the most numerous and cosmopolitan marine bacterioplankton clades and, ultimately, to determine the functional traits that make them so successful [10–12]. More recently, high-throughput sequencing technologies have allowed for increasing the depth of investigation and thereby unveiled a rare biosphere that accounts for most of the observed phylogenetic diversity of bacterioplankton community [13,14]. This acts as a “seed bank” from where new dominant species can emerge when the environmental conditions change [4,15].

Consequently, species-sorting by the local environment has been demonstrated to be one of the main driving processes behind shaping the BCC [16–19]. However, in some cases, the assembly mechanism can be well explained by neutral models [20–23], by mass effects [24,25], or by the combination of several mechanisms [26–28]; the relative importance of these mechanisms may change over time [29].

In addition to unique environmental conditions, similarities to other communities have to be considered in order to identify processes underlying the assembly of local microbial communities [23]. Hence, in this study, we combined environmental factors with phylogenetic affiliations of relatively abundant populations for that purpose. Hence, special attention was paid to associations within between ribotypes, because these interactions can have stronger correlative relationships compared to relationships between bacteria and eukaryotes, or bacteria and abiotic environmental factors [30]. The co-occurrence of networks of dominant bacterial ribotypes isolated from the marine oxygen minimum zone (OMZ) throughout the world has revealed a pattern of cosmopolitan key species filling redox-driven niches [31]. These niches revolve around functional capabilities to utilize different electron receptors and donors [32]. Next important step towards a better understanding of these microbial communities inhabiting OMZ is to define shared or specialized metabolic subsystems in different oceanic provinces [31]. Our results contribute to this effort.

The Baltic Sea is one of the largest brackish basins of the world, characterized by a long residence time. Therefore, it is not just a mixing zone for fresh water and marine species, but a habitat for microbes specialized for brackish water, which has been illustrated by the spread of different bacterial populations throughout the salinity gradient of the Baltic Sea [33–35]. Unlike the diversity of macro-organisms, the BCC does not decline within a salinity gradient [35].

The Gulf of Finland is the easternmost sub-basin of the Baltic Sea. The strong stratification in the central part of the gulf due to the seasonal thermocline and permanent halocline often hinders mixing in the water column [36]. Eutrophication-driven phytoplankton production leads to increased sedimentation of organic matter and hence increased consumption of oxygen for which atmospheric and photosynthetic re-oxygenation cannot compensate [37].

Furthermore, the Gulf of Finland is directly connected to the Baltic Proper, where the anoxic zone is permanent and therefore inhabited by well-established anaerobic ecotypes typical of the Baltic Proper [36,38,39]. During oxygen deficiency, certain microbes are capable of using terminal electron acceptors other than oxygen (e.g. NO_3_^-^, SO_4_^2-^ and metal oxides). Epsilonproteobacterial *Sulfurimonas gotlandica* clade GD1 has been demonstrated to be one of the most numerous chemolithoautotrophic bacteria present in the OMZ of the central Baltic Sea [40,41]. This clade has been shown to spread into the anoxic zone of the Gulf of Finland [42].

As a temperate estuary, the Gulf of Finland undergoes many seasonal changes in environmental parameters such as ice coverage, water temperature, solar radiance, inorganic nutrients, etc., which contribute to the recurring succession of phytoplankton. Variation in biotic and abiotic factors also leads to the recurring succession of bacterioplankton in the Baltic Sea [34,43,44].

The goals of present study were to investigate the spatial and temporal dynamics of the BCC of the Gulf of Finland during the spring to summer transition in order to determine the main factors driving the bacterioplankton community assembly. To these ends, we used pyrosequencing of 16S rRNA genes from community DNA samples, which were collected in parallel with monitoring of physicochemical parameters and phytoplankton community composition. Many annually recurring environmental shifts took place during the study period, mainly the formation of a thermocline, the depletion of oxygen in the deeper layers and the shift from the spring to summer phytoplankton community.

## Materials and Methods

### Ethics statement

No specific permits were required for the described field studies. Our study area is not privately owned or protected in any way. The study did not involve endangered or protected species.

### Sample collection and extraction of community DNA

**Fig 1:**
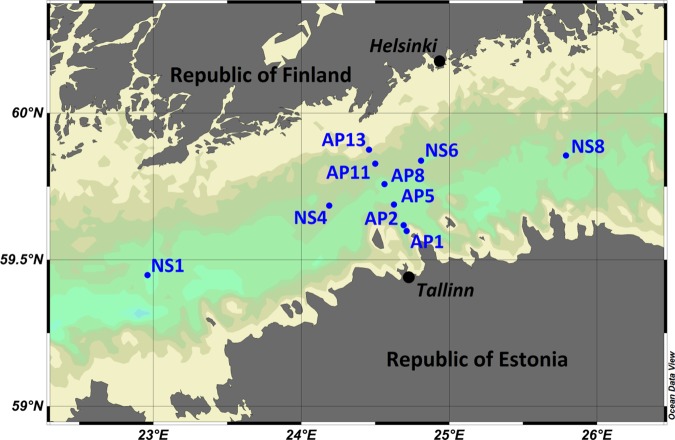
Map of the study area with sampling stations.

Water sampling was performed aboard the RV Salme in the spring and summer of 2011 on two transects in the central part of the Gulf of Finland (Fig 1). The coordinates of sampling stations are listed in Table 1. Samples were collected from three depths: at 5 m, 40 m and about 5 m above the seafloor; detailed information about each sample is provided in Table 2. A rosette sampler (M1018, General Oceanics) equipped with Niskin water samplers (volume 1.7 l) was used for sampling. For background information, the depth profiles of conductivity and temperature were obtained with a SBE19plus CTD probe (conductivity-temperature-depth probe, Sea-Bird Electronics), chlorophyll *a* fluorescence with WETStar fluorometer (WETLabs) and dissolved oxygen with SBE 43 probe (Sea-Bird Electronics).

**Table 1:** Coordinates of the sampling stations.

Station	Longitude	Latitude
AP1	24.71337	59.59792
AP2	24.69253	59.61823
AP5	24.62698	59.68858
AP8	24.56268	59.75820
AP11	24.50003	59.82780
AP13	24.45807	59.87580
NS1	22.96157	59.44745
NS4	59.68333	24.18667
NS6	24.81060	59.83822
NS8	25.78967	59.85605

**Table 2:** The physico-chemical properties of the sampling sites. NA–not analyzed.

Code	Date	Station	Depth (m)	Volume (ml)	Oxygen (mg/L)	Temperature (°C)	Chl_a (mg m^-3^)	Salinity	NO2/NO3 (μmol/L)	PO4 (μmol/L)
**WA_000034**	**21.04.2011**	**AP2**	**5**	**750**	**16.0**	**0.8**	**5.8**	**6.4**	**0.1**	**0.8**
**WA_000035**	**21.04.2011**	**AP2**	**40**	**600**	**14.7**	**0.1**	**1.8**	**6.8**	**5.8**	**1.2**
**WA_000036**	**21.04.2011**	**AP2**	**95**	**750**	**1.7**	**4.9**	**0.2**	**9.5**	**4.8**	**2.5**
**WB_000038K**	**21.04.2011**	**AP5**	**40**	**900**	**13.8**	**0.5**	**0.4**	**6.9**	**6.0**	**1.1**
**WA_000039**	**21.04.2011**	**AP5**	**83**	**700**	**1.4**	**5.1**	**0.2**	**9.7**	**4.7**	**2.9**
**WA_000040**	**21.04.2011**	**AP8**	**5**	**700**	**15.5**	**0.9**	**6.8**	**6.5**	**0.1**	**0.7**
**WB_000040K**	**21.04.2011**	**AP8**	**5**	**700**	**15.5**	**0.9**	**6.8**	**6.5**	**0.1**	**0.7**
**WB_000041K**	**21.04.2011**	**AP8**	**40**	**850**	**15.3**	**0.1**	**0.6**	**6.7**	**3.9**	**0.8**
**WB_000042K**	**21.04.2011**	**AP8**	**76**	**900**	**4.7**	**4.0**	**0.2**	**8.7**	**4.3**	**1.8**
**WB_000043K**	**4.05.2011**	**AP2**	**5**	**800**	**13.9**	**1.6**	**8.6**	**6.1**	**1.2**	**0.6**
**WA_000044**	**4.05.2011**	**AP2**	**41**	**950**	**9.3**	**1.5**	**0.6**	**7.2**	**4.5**	**1.2**
**WA_000049**	**4.05.2011**	**AP8**	**5**	**500**	**15.0**	**2.4**	**4.5**	**6.3**	**0.0**	**0.7**
**WA_000054**	**4.05.2011**	**AP13**	**5**	**750**	**14.7**	**3.1**	**5.7**	**6.2**	**0.3**	**0.6**
**WA_000057**	**10.05.2011**	**NS1**	**40**	**1000**	**14.5**	**1.2**	**3.1**	**7.0**	**NA**	**NA**
**WA_000058**	**10.05.2011**	**NS1**	**80**	**950**	**0.1**	**5.7**	**0.2**	**10.4**	**NA**	**NA**
**WA_000059**	**10.05.2011**	**NS4**	**5**	**650**	**16.0**	**3.2**	**4.2**	**6.1**	**NA**	**NA**
**WB_000060**	**10.05.2011**	**NS4**	**40**	**1000**	**14.1**	**1.3**	**2.2**	**6.9**	**NA**	**NA**
**WB_000061**	**10.05.2011**	**NS4**	**63**	**950**	**5.9**	**3.4**	**0.9**	**8.5**	**NA**	**NA**
**WB_000062**	**10.05.2011**	**NS6**	**5**	**750**	**15.8**	**5.5**	**4.4**	**5.9**	**NA**	**NA**
**WB_000063**	**10.05.2011**	**NS6**	**40**	**1000**	**12.8**	**0.5**	**1.7**	**6.7**	**NA**	**NA**
**WB_000064**	**10.05.2011**	**NS6**	**70**	**1000**	**2.9**	**4.5**	**0.5**	**9.2**	**NA**	**NA**
**WA_000065**	**10.05.2011**	**NS8**	**5**	**800**	**15.1**	**0.6**	**12.5**	**4.8**	**NA**	**NA**
**WA_000066**	**10.05.2011**	**NS8**	**40**	**850**	**9.3**	**1.9**	**1.5**	**7.0**	**NA**	**NA**
**WB_000068**	**3.06.2011**	**NS6**	**5**	**750**	**12.3**	**7.9**	**1.1**	**5.9**	**NA**	**NA**
**WB_000069**	**3.06.2011**	**NS6**	**40**	**850**	**11.2**	**0.8**	**0.7**	**6.8**	**NA**	**NA**
**WB_000070**	**3.06.2011**	**NS6**	**72**	**1000**	**2.5**	**3.7**	**0.4**	**8.7**	**NA**	**NA**
**WA_000072**	**3.06.2011**	**NS8**	**40**	**950**	**10.6**	**1.3**	**0.6**	**6.9**	**NA**	**NA**
**WB_000073**	**3.06.2011**	**NS8**	**80**	**1000**	**2.0**	**4.3**	**0.3**	**8.9**	**NA**	**NA**
**WA_000082**	**3.06.2011**	**NS4**	**5**	**750**	**11.3**	**9.5**	**1.0**	**6.3**	**NA**	**NA**
**WB_000085**	**3.06.2011**	**NS1**	**5**	**800**	**11.6**	**9.4**	**0.5**	**6.6**	**NA**	**NA**
**WB_000086**	**3.06.2011**	**NS1**	**40**	**950**	**10.9**	**1.1**	**0.2**	**7.0**	**NA**	**NA**
**WB_000087**	**3.06.2011**	**NS1**	**84**	**1000**	**0.5**	**5.1**	**0.2**	**9.7**	**NA**	**NA**
**WB_000090**	**10.06.2011**	**AP2**	**40**	**800**	**11.6**	**2.2**	**0.2**	**7.0**	**1.1**	**0.6**
**WB_000091**	**10.06.2011**	**AP2**	**90**	**800**	**0.2**	**5.0**	**0.2**	**9.6**	**1.3**	**2.6**
**WB_000093**	**10.06.2011**	**AP5**	**40**	**850**	**12.8**	**2.8**	**0.2**	**6.9**	**0.4**	**0.3**
**WB_000095**	**10.06.2011**	**AP8**	**5**	**550**	**10.7**	**12.8**	**1.0**	**6.4**	**0.0**	**0.2**
**WB_000098**	**10.06.2011**	**AP11**	**5**	**600**	**10.9**	**12.6**	**1.2**	**6.4**	**NA**	**NA**
**WB_000100**	**10.06.2011**	**AP13**	**5**	**775**	**12.1**	**10.6**	**1.1**	**6.1**	**0.0**	**0.3**
**WB_000101**	**10.06.2011**	**AP13**	**31**	**900**	**12.2**	**4.0**	**0.5**	**6.4**	**0.3**	**0.6**
**WB_000102**	**14.07.2011**	**AP1**	**5**	**625**	**10.0**	**16.9**	**1.2**	**6.3**	**NA**	**NA**
**WB_000103**	**14.07.2011**	**AP2**	**5**	**850**	**10.0**	**17.1**	**1.9**	**6.2**	**0.0**	**0.2**
**WB_000106**	**14.07.2011**	**AP5**	**5**	**600**	**10.4**	**17.7**	**1.9**	**5.6**	**0.0**	**0.2**
**WB_000107**	**14.07.2011**	**AP5**	**40**	**725**	**12.5**	**3.3**	**0.2**	**6.9**	**1.0**	**0.8**
**WA_000108**	**14.07.2011**	**AP5**	**84**	**850**	**0.2**	**5.0**	**0.2**	**9.7**	**0.2**	**4.4**
**WB_000109**	**14.07.2011**	**AP8**	**5**	**500**	**10.5**	**18.7**	**5.1**	**6.0**	**0.0**	**0.3**
**WB_000110**	**14.07.2011**	**AP8**	**40**	**800**	**12.6**	**4.0**	**0.2**	**6.8**	**0.6**	**0.9**
**WB_000111**	**14.07.2011**	**AP8**	**74**	**900**	**2.7**	**3.9**	**0.2**	**8.8**	**5.3**	**3.5**
**WB_000112**	**14.07.2011**	**AP11**	**5**	**675**	**10.1**	**18.5**	**4.0**	**6.2**	**NA**	**NA**

The samples for nutrient (NO_2_^−^ + NO_3_^−^ and PO_4_^3−^) analysis were deep-frozen at −20°C after collection and analyzed at a shore-based laboratory using a Lachat QuikChem 8500 Series 2 automatic nutrient analyzer (Lachat Instruments, Hach Company). The nutrient analyses were performed according to the ISO 15681–1 method for PO_4_^3−^ and ISO 13395 method for NO_2_^−^ + NO_3_^−^. The lower detection ranges for PO_4_^3−^ and NO_2_^−^ + NO_3_^−^ were 0.02 and 0.03 μmol l^-1^, respectively.

Water samples for microbial community extraction were collected into sterile bottles (Nalgene) and immediately filtered through 0.2 μm filters (Whatman, Puradisc FP 30) after preliminary filtration through 5.0 μm prefilters (Whatman, Puradisc FP 30). The scheme of the filtration system was described by Laas *et al*. (2014) [42]. The sample volume varied between 0.5 and 1.0 liters. Filters were kept frozen at -20°C until community DNA was extracted with a PowerSoil DNA Isolation Kit (MO BIO Laboratories, Inc.). A few modifications were made to the protocol: syringe filters were incubated with the lysis buffer in the casing at 60°C for 30 min and then the eluate was removed.

### Amplification of bacterial 16S rRNA gene sequences

The bacterial 16S rRNA gene V1-V2 hypervariable regions were amplified in two polymerase chain reactions (PCR). For the first reaction, universal bacterial primers BSF8 and BSR357 were complemented with 8 nt barcode and partial adapter sequences (Table 3) [46]. PCR was performed with Smart-Taq Hot Red 2X PCR Mix (Naxo, Estonia), 1 μl of extracted DNA and 0.2 μM each primer, using the following cycling parameters: 15 min denaturation followed by three cycles (30 sec at 95°C, 30 sec at 50°C, 60 sec at 72°C), 28 cycles (30 sec at 95°C, 30 sec at 65°C, 60 sec at 72°C) and a final extension at 72°C for 7 min. To achieve full length sequencing adapters, second PCR amplification was performed.

**Table 3:** Primers used in this study.

Primer name	Sequence 5'- 3'	Citation
F8	TTGGCAGTCTCAGnnnnnnnnAGTTTGATCCTGGCTCAG*	[45]
R357	GTCTCCGACTCAGnnnnnnnnCTGCTGCCTYCCGTA*	[45]
Adapter A	CCATCTCATCCCTGCGTGTCTCCGACTCAG	**
Adapter B	CCTATCCCCTGTGTGCCTTGGCAGTCTCAG	**

*—nnnnnnnn is the barcode

**—standard adapter for 454 Titanium chemistry

The second reaction was run with Smart-Taq Hot Red 2X PCR Mix (Naxo, Estonia), 1 μl of 10 X diluted amplicon, 0.2 μM each primer; using the following cycling parameters: 15 min denaturation followed by five cycles (30 sec at 95°C, 30 sec at 62°C, 60 sec at 72°C), 20 cycles (30 sec at 95°C, 60 sec at 72°C) and a final extension at 72°C for 10 min. PCR reactions were run on a thermal cycler (model 2720, Applied Biosystems). Each PCR product was gel purified on a 1.5% agarose gel. DNA was isolated using the QIAquick Gel extraction kit (Qiagen, Inc.). DNA concentrations were measured with a Qubit fluorometer (Invitrogen). Sequencing was performed on a Roche GS FLX next generation sequencing platform (IMGM Laboratories).

### Bioinformatics and statistics

Reads with low quality and those shorter than 150 bp (basepairs) were removed from the dataset. The PyroNoise algorithm was used to discard homopolymer-derived errors [47] and UCHIME to remove chimeric DNA sequences caused by PCR errors [48]. OTUs (Operational Taxonomic Units) were defined using the average neighbor-clustering algorithm of MOTHUR 1.19.1 [49] with a 97% similarity threshold. Reference sequences were selected from the SILVA ribosomal RNA database [50]. Taxonomic assignments were processed by the Ribosomal Database Project (RDP) naïve Bayesian Classifier [51]. For database affiliations RDP Seqmatch [52] and BLAST [53] search algorithms were used against RDP and a NCBI (National Center for Biotechnology Information) nucleotide databases, respectively. Chloroplast and mitochondrial sequences were discarded from the dataset. Statistical analyses were carried out with the R program version 2.14.0 (http://www.r-project.org), ACE (Abundance-based Coverage Estimation) [54] and Chao1 [55] richness estimates; multivariate statistics were calculated using the VEGAN package [56]. The similarity matrices and clustering were generated using the gplots package [57]. The sequences have been deposited into GenBank (accession numbers from KM489611 to KM491167).

## Results

### Environmental parameters structuring the bacterial community

**Fig 2:**
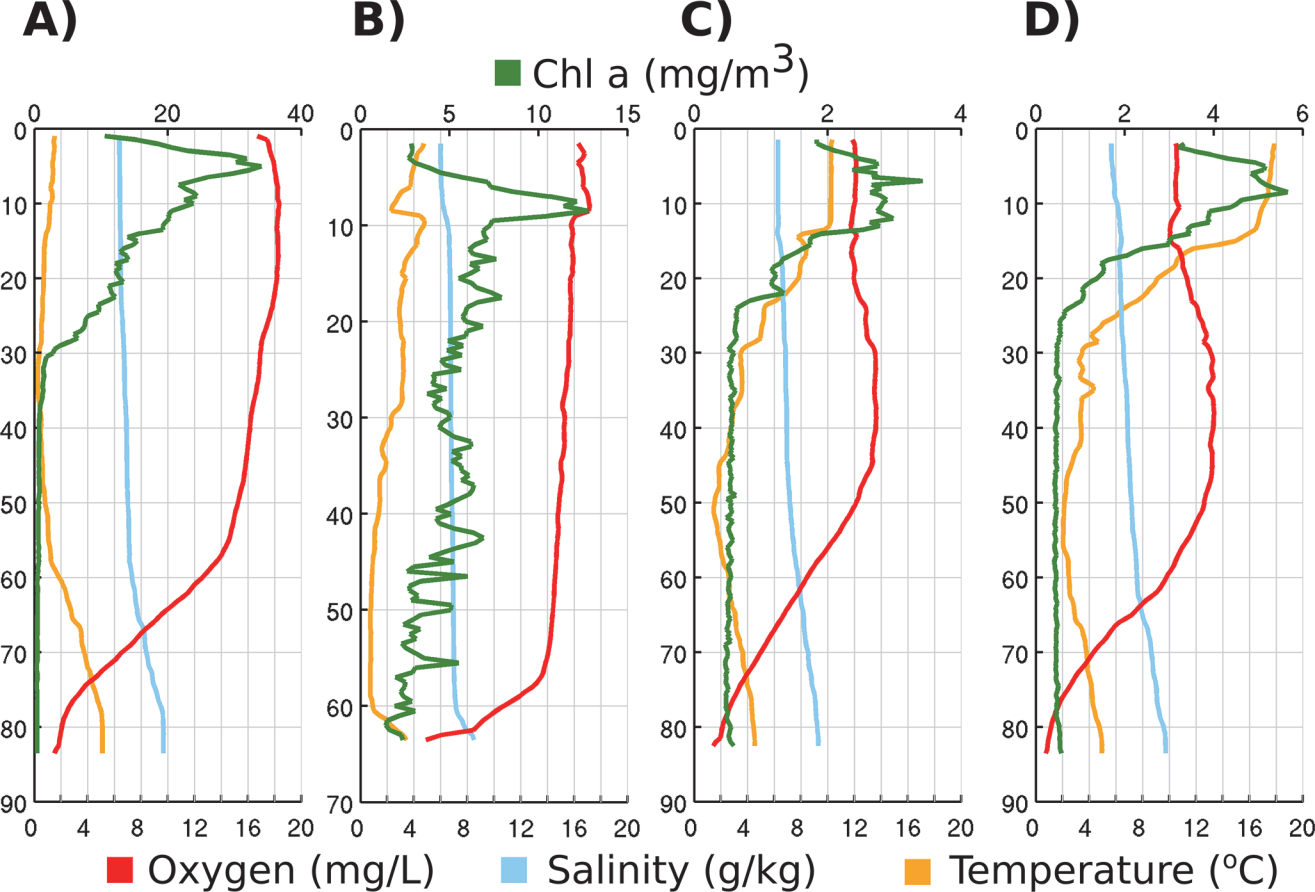
Water column profiles of station AP5 on 21th April (A), on 10th June (C), on 14th July (D) and station NS4 on 10th May (B).

The sampling took place from April to July in 2011 and the bacterial 16S rRNA gene libraries were generated from three sampling depths: (i) surface water at 5 m, (ii) the intermediate layer at 40 m and (iii) the near-bottom layer a few meters above the seafloor; depth varied between stations (63–95 m, Table 2). These depths were chosen because, in summer, the gulf is stratified into three layers. Four hydrographic profiles are visualized in Fig 2 to provide overview of changes of environmental parameters in the water column throughout the sampling period.

The 5 m and 40 m horizons always remained above the permanent halocline and at those depths the salinity varied in the range of 4.8–6.6 g kg^-1^ and 6.7–7.2 g kg^-1^, respectively. Salinity decreased from west to east, with the lowest value recorded in the easternmost surface sample. The near-bottom layer samples were collected at the halocline or below it and the salinity ranged from 8.5–10.4 g kg^-1^. The temperature varied little in the near-bottom layer (3.7–5.0°C in April and 0.1–4.0°C in July), but at the 5 m depth, warming was observed from 0.8–0.9°C in April to 17.1–18.7°C in July. The most remarkable change in temperature occurred between May and June, when a well-pronounced thermocline started to form at 10–25 m.

Oxygen conditions were hypoxic or anoxic in deeper sampling layer (> 63 m) throughout the sampling period. Oxygen concentrations at the 5 m depth were significantly (p < 0.01, Student’s t-test) lower during the summer (10.0–12.3 mg l^-1^) compared with the spring (13.9–16.0 mg l^-1^). At 40 m depth, the overall tendency remained similar to the upper layer, but on two occasions in May, the oxygen concentrations were below 9.5 mg l^-1^.

Chlorophyll *a* concentrations were highest in April (4.2–12.5 mg m^-3^, Table 2), when phytoplankton was dominated by *Diatomphyceae* (data not shown). Also, concentrations of inorganic nutrients at 5 m depth we higher during the spring months compared with June or July. Nitrites and nitrates remained at concentration levels from the detection limit to 1.2 μmol l^-1^ and phosphates varied between 0.6–0.8 μmol l^-1^. In June-July, the concentrations of nitrites and nitrates remained below the detection limit and phytoplankton was dominated by diazotrophic *Nostocophyceae* (data not shown). The concentration of phosphates was also lower in the surface layer during the summer months (Table 2).

### Bacterioplankton community diversity

A total of 73,494 partial rRNA gene sequences and 48 different samples were used in this analysis, on average 2,161 sequences per sample. The minimum number of sequences per sample was set to 350. In Table 2, the number of observed OTUs is accompanied with the Chao1 and ACE species richness estimates for each sample. Overall, the number of OTUs was significantly higher in the near-bottom layer (on average 144, SD = 34) than at 5 m and 40 m (98, SD = 44 and 90, SD = 43; respectively). Based on the rarefaction curves outlined from samples (S1 Fig), we assume that deeper sequencing (more sequences per sample) would have resulted in significantly higher estimates. Therefore, in this study, we could not study rare species and therefore concentrated on abundant members of the bacterioplankton community.

### Bacterioplankton community composition

A total of 29 different bacterial classes were found. More than 85% of identified OTUs belonged to eight classes: *Alphaproteobacteria* (31.2%), *Actinobacteria* (17.8%), *Betaproteobacteria* (8.9%), *Cyanobacteria* (9.3%), *Epsilonproteobacteria* (5.8%), *Gammaproteobacteria* (2.5%), *Flavobacteria* (8.7%) and *Sphingobacteria* (1.6%). About 1/10 (11.6%) of the sequences remained unclassified at the bacterial class level. A total of 1,557 OTUs (97% cutoff) were obtained, out of which 839 were single-read OTUs. The most numerous OTUs with their database affiliations are listed in Table 4.

**Table 4:** Database affiliations of the dominant OTUs (>300 sequences in the entire dataset).

OUT classification	OTU code	Isolation source (RDP)	Accession nr. Genbank (RDP)	%	Isolation source (BLAST)	Accession nr. Genbank (BLAST)	%
**Bacteroidetes(100)**	**BSNS3145**	**deep-sea octacoral**	**DQ395498**	**90.9**	**coastal ocean**	**KC336890**	**99**
**Actinomycetales(100)**	**BSNS3157**	**lake epilimnion (Jimi Hendrix Bog Lake**	**EU117758**	**100**	**Gulf of Gdansk (Baltic Sea), 1 m**	**KF596574**	**100**
**Actinomycetales(100)**	**BSNS3107**	**lake epilimnion**	**EU117608**	**100**	**river water**	**GU641290**	**100**
**Flavobacteriaceae(100)**	**BSNS3079**	**marine biome, fjord, coastal water**	**FR686324**	**99.6**	**coastal ocean**	**KC336902**	**100**
**Rhodobacteraceae(100)**	**BSNS3149**	**culture collection: ATCC:17025**	**CP000661**	**97**	**marine bulk water**	**JX015552**	**100**
**Flavobacteriaceae(100)**	**BSNS3078**	**Lake Zurich, Spring Bloom 2009**	**HE574367**	**92.1**	**seawater, 2 m depth**	**FR648023**	**99**
**Bacteroidetes(100)**	**BSNS3163**	**coastal water**	**GU230419**	**94.8**	**Baltic Sea, 3m depth, Landsort deep St. BY31**	**EF627875**	**99**
**Polaribacter(100)**	**BSNS3072**	**Arctic sea water**	**EU330381**	NC*	**Arctic Sea water**	**FJ196065**	**99**
**Actinomycetales(100)**	**BSNS3110**	**lake epilimnion**	**EU117955**	**99.6**	**off-seep, ice-free (Alaska)**	**JN626833**	**99**
**Microbacteriaceae(100)**	**BSNS2531**	**lake water, West Lobe at 13 m**	**DQ015847**	**98.4**	**Arctic Ocean**	**JN976695**	**100**
**Flavobacteriaceae(100)**	**BSNS2870**	**oil-contaminated seawater**	**JQ712124**	**94.6**	**genomic DNA**	**AF388893**	**99**
**Comamonadaceae(100)**	**BSNS3044**	**mangrove**	**DQ234161**	**99.6**	**coastal ocean**	**KC336502**	**100**
**Pelagibacter(100)**	**BSNS3084**	**Chesapeake Bay, 8 m**	**EU800103**	**99.2**	**Gulf of Gdansk (Baltic Sea), 1 m**	**KF596598**	**99**
**Pelagibacter(100)**	**BSNS3076**	**deep-sea octacoral**	**DQ395535**	**100**	**300m depth water sample**	**JX530818**	**100**
**Pelagibacter(100)**	**BSNS3171**	**Chesapeake Bay, 25 m**	**EU801724**	**98.8**	**Qinghai Lake**	**HM127540**	**99**
**Bacteria(100)**	**BSNS3058**	**marine biome, fjord, coastal water**	**FR683807**	**96.2**	**surface water in the Northern Bering Sea**	**GQ452877**	**100**
**Ilumatobacter(100)**	**BSNS3154**	**Chesapeake Bay, 25 m**	**EU802230**	**100**	**Gulf of Gdansk (Baltic Sea), 1 m**	**KF596602**	**100**
**Flavobacteriales(99)**	**BSNS2920**	**Chesapeake Bay, 25 m**	**EU802220**	**97.6**	**genomic DNA**	**AF388893**	**99**
**Ilumatobacter(100)**	**BSNS2659**	**Delaware Bay, 8 m**	**EU800747**	**99.6**	**water of common carp culture pond**	**JQ305072**	**100**
**GpIIa(100)**	**BSNS2840**	**Synechococcus sp. isolate**	**AY151241**	**100**	**the Gulf of Finland (The Baltic Sea)**	**FR820441**	**100**
**Rhodothermaceae(100)**	**BSNS3172**	**deep-sea sediments**	**AB015587**	**95.5**	**Crambe crambe (sponge)**	**GU799618**	**98**
**Bacteria(100)**	**BSNS3174**	**marine sediment**	**FJ813551**	**93.6**	**municipal activated sludge wastewater treatment bioreactor**	**HQ509575**	**93**
**Rhodobacteraceae(100)**	**BSNS3179**	**ocean water from the Yellow Sea**	**HM057611**	**98.1**	**Crambe crambe (sponge)**	**GU799618**	**98**
**Alphaproteobacteria(100)**	**BSNS3143**	**strain of Reyranella massiliensis**	**EF394922**	**100**	**drinking water**	**KF515019**	**100**
**Gammaproteobacteria(100)**	**BSNS3168**	**Baltic Sea redoxcline, 119 m depth**	**JX974825**	**98.7**	**marine sample**	**JQ859404**	**99**
**Flavobacteriaceae(100)**	**BSNS3178**	**Baltic Sea redoxcline, 119 m depth**	**KC492867**	**100**	**Baltic Sea redoxcline, 119 m depth**	**KC492867**	**99**
**Methylobacter(100)**	**BSNS3164**	**cultured strain**	**AF152597**	**100**	**groundwater discharge zone sediment**	**KC922589**	**100**
**Ilumatobacter(100)**	**BSNS3169**	**hydrothermal vent waters**	**HM446118**	**99.6**	**Lubomirskia baicalensis (freshwater sponge)**	**JQ272709**	**100**
**Methylophilus(97)**	**BSNS3170**	**Kerguelen Plateau in the Southern Ocean, 120 m**	**EU005833**	**98.2**	**ocean surface water**	**JQ253996**	**99**
**Actinomycetales(100)**	**BSNS3032**	**deep-sea octacoral**	**DQ396268**	**99.6**	**Saanich Inlet, 10 m depth**	**GQ346797**	**99**
**Actinomycetales(100)**	**BSNS3133**	**bottom water in the northern Bering Sea**	**GQ850562**	**99.6**	**Saanich Inlet, 10 m depth**	**GQ346797**	**99**
**Actinomycetales(100)**	**BSNS3120**	**deep-sea octacoral**	**DQ396268**	**99.2**	**whole surface water from Chesapeake Bay**	**EF471727**	**100**
**Bacteroidetes(100)**	**BSNS3126**	**Baltic Sea redoxcline, 119 m depth**	**KC492874**	**100**	**Baltic Sea redoxcline, 119 m depth**	**KC492874**	**99**
**Sulfurimonas(100)**	**BSNS3177**	**Baltic Sea redoxcline, 119 m depth**	**KC492833**	**99.6**	**Baltic Sea redoxcline, 119 m depth**	**KC492833**	**100**
**Solirubrobacterales(100)**	**BSNS3175**	**soil sample above gas and oil field**	**GU056099**	**98.4**	**Baltic Sea brackish sediment, depth 0–1 cm**	**FN423884**	**99**
**Corynebacterineae(100)**	**BSNS3016**	**Lake Pavin (meromictic lake)**	**GU472705**	**96.9**	**the Gulf of Finland (The Baltic Sea) sediment**	**FR820412**	**100**

Best matches found with the RDP Seqmatch tool and BLAST against the NCBI nucleotide database are listed and accompanied by the corresponding isolation source.

**Fig 3:**
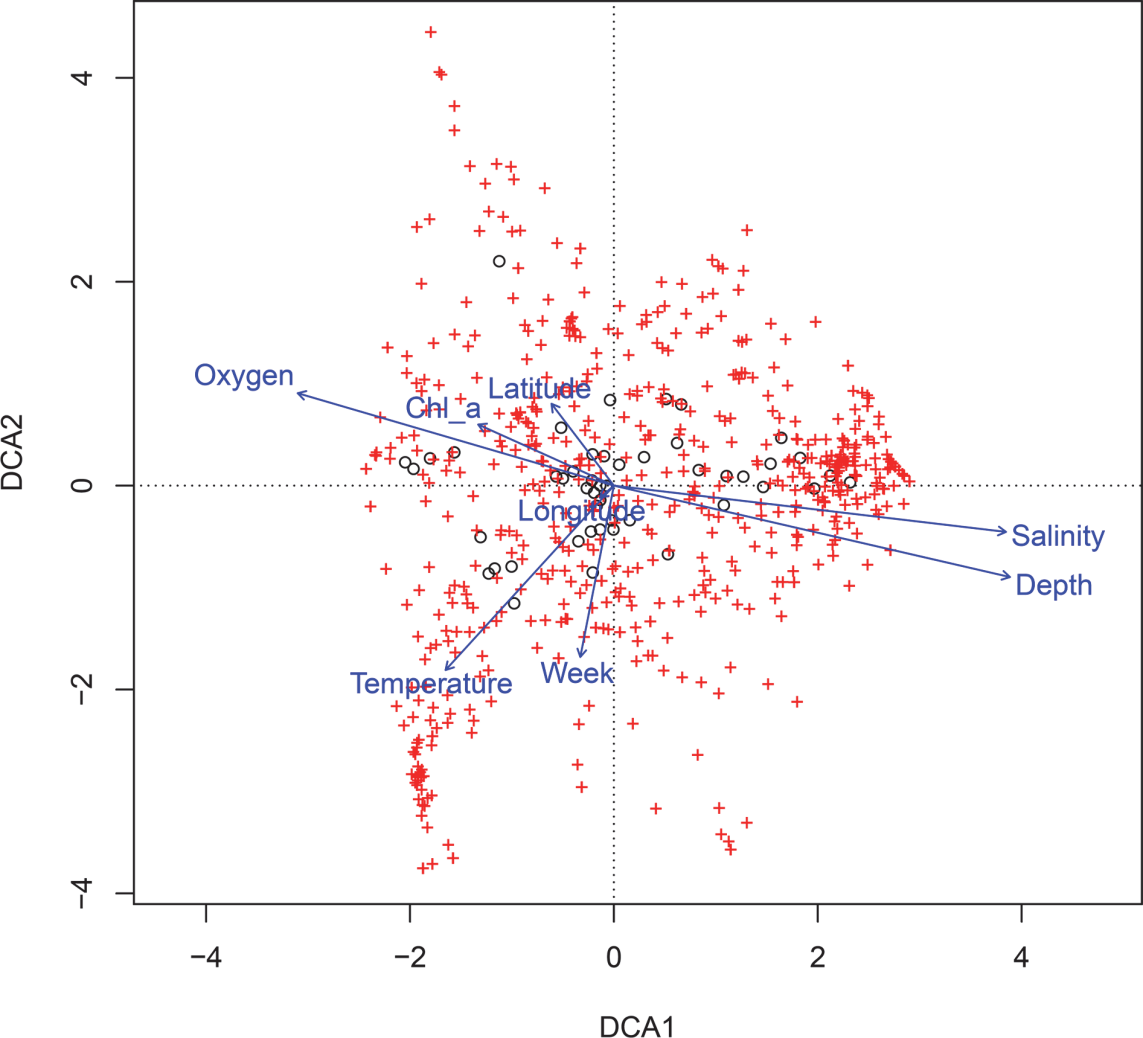
Detrended correspondence analysis of the bacterioplankton community composition on the operational taxonomic unit level (97% similarity). Axis 1 and axis 2 explain 11.1% and 9.5% of the variation, respectively.

**Fig 4:**
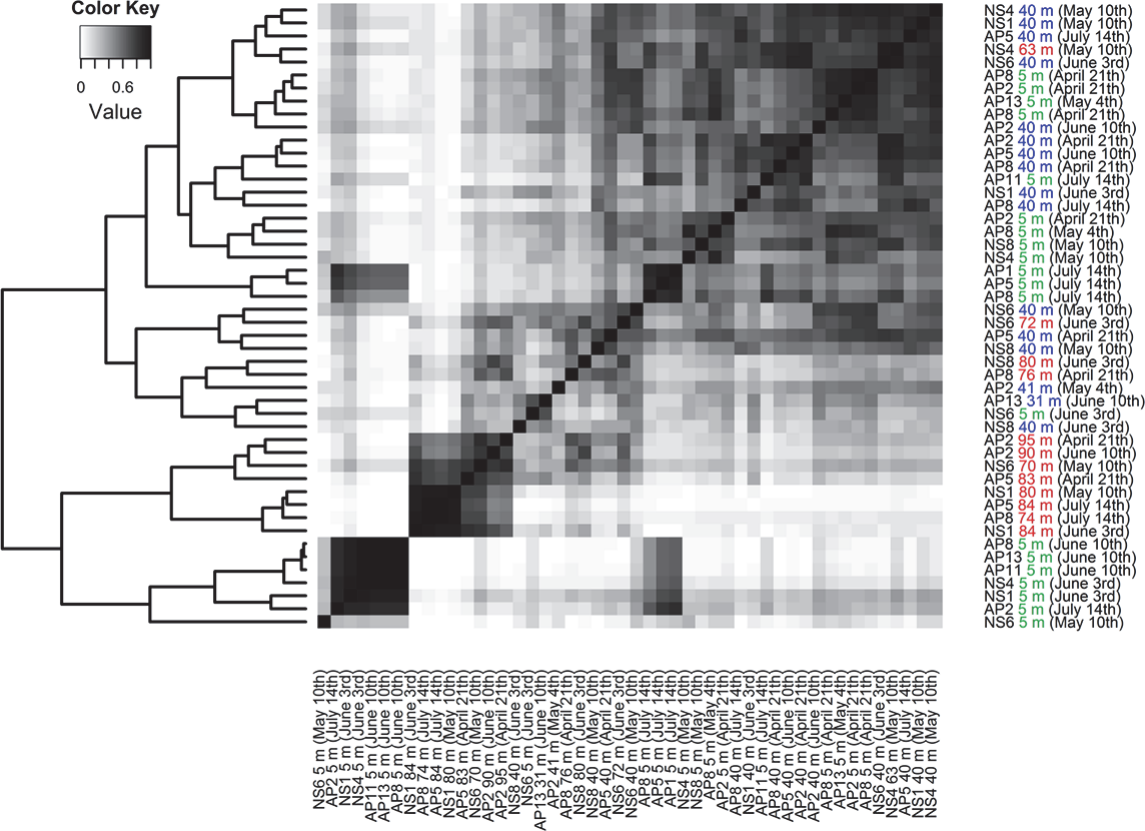
Pair-wise similarity matrix of the bacterial community based on relative abundances of OTUs. The dendrogram represents clustering based on pair-wise similarities in r-values (Pearson). Three different sampling depths are color-coded green (5 m), blue (30–40 m) and red (near-bottom).

**Fig 5:**
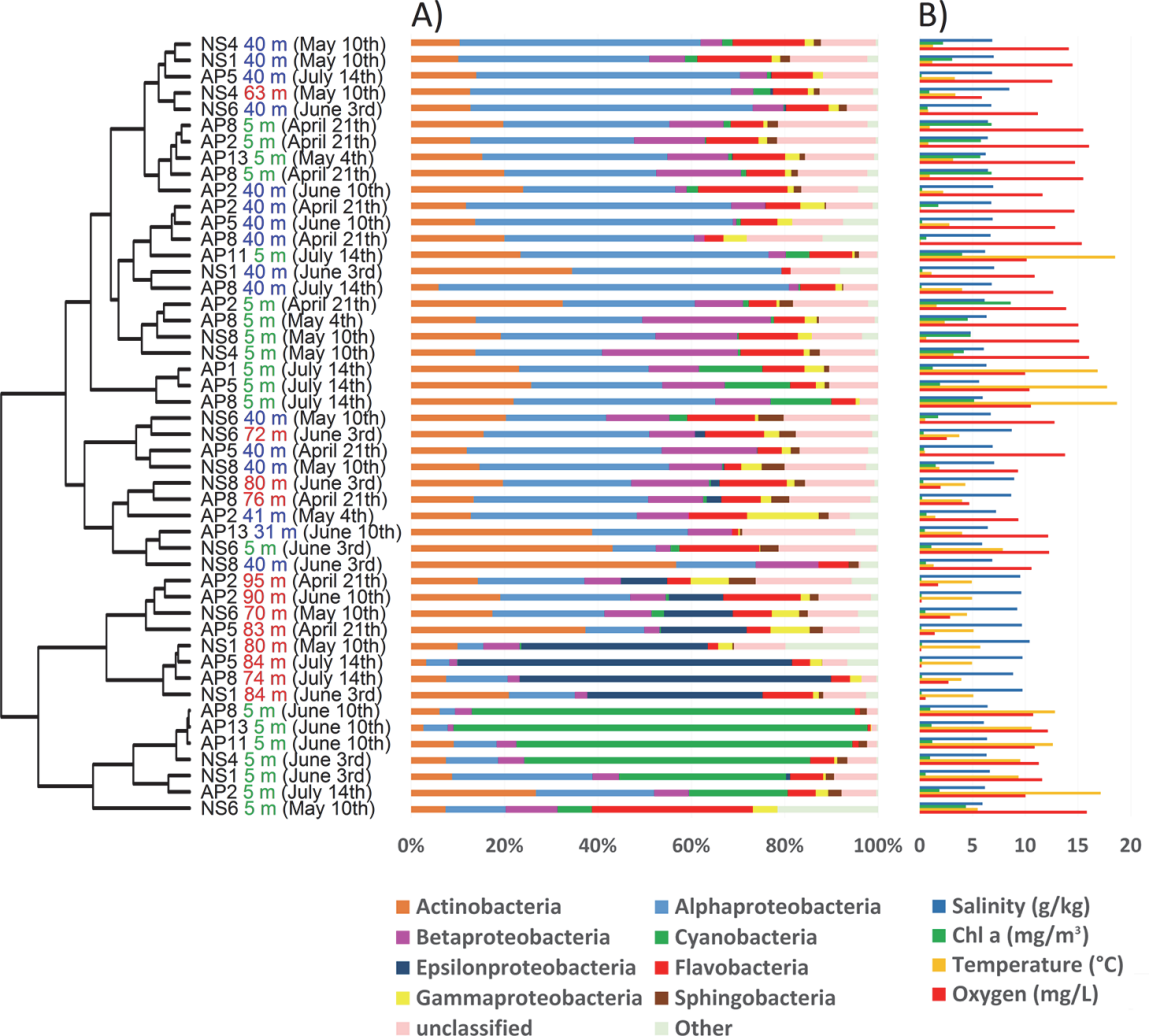
The relative abundance of the eight most abundant bacterial classes and a pooled group of unclassified ribotypes (A) that are accompanied with environmental parameters (B). The dendrogram is adopted from Fig 3.

**Fig 6:**
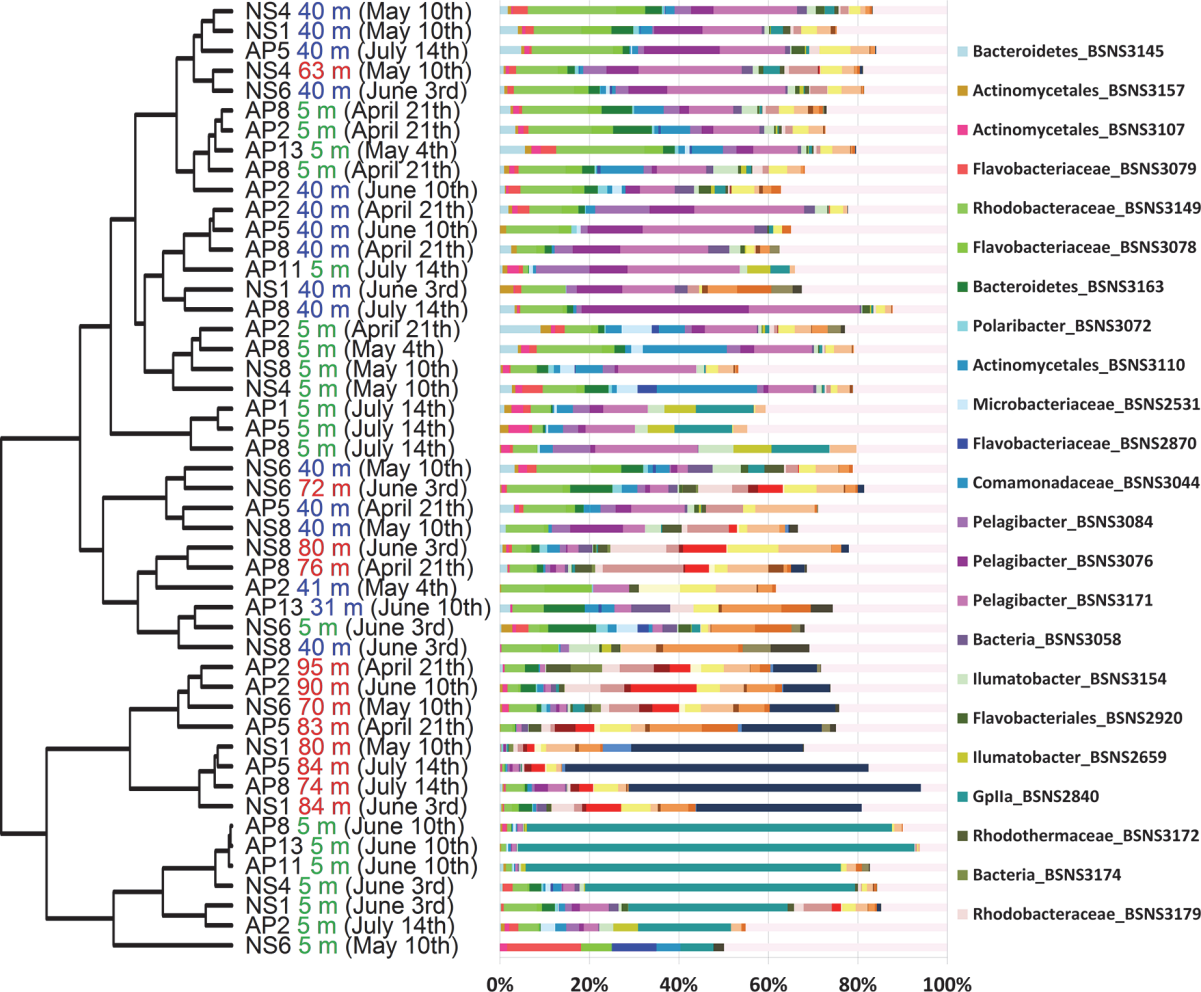
The relative abundance of dominant OTUs (>300 sequences in the entire dataset). The dendrogram is adopted from Fig 3.

A descriptive multivariate statistical method, i.e. detrended correspondence analysis (DCA), was used to describe the overall OTU abundance and thereafter a vector-fitting procedure was applied to determine which environmental variables significantly related to BCC patterns (Fig 3, Table 5). As a result, oxygen, depth and salinity were distinguished as the most important co-varying environmental factors (r^2^ and p values are presented in Table 5). Temperature, sampling time and Chl α could be identified as being less significant to the DCA space (in declining order). The geographic location (longitude and latitude) was rendered non-significant. The DCA also indicates how measured environmental parameters co-varied. To further investigate the dynamics of BCC, Pearson correlation analysis was carried out based on the relative abundance of OTUs, which was used to construct a similarity matrix (Fig 4). Organized according to clustering of the similarity matrix, all community profiles are visualized on the class level in Fig 5 for an overview and the OTU level (97% similarity) in Fig 6 to provide detailed insight into the relative abundance of dominant ribotypes. On a broader scale, the communities were divided into three groups: (i) surface communities during the summer months that were dominated by the unicellular cyanobacterium *Synechococcus* (OTU BSNS2840); (ii) hypoxic/anoxic near-bottom communities that contained a large fraction of chemolithotrophic *Sulfurimonas* (OTU BSNS3177); and (iii) a larger group with all remaining communities with notable subdivisions among them.

**Table 5:** Results of the detrended correspondence analysis of the bacterioplankton community composition.

	DCA1 (11.1%)	DCA2 (9.5%)	r^2^	Pr(>r)
Oxygen	-0.95981	0.28064	0.4942	0.000999
Depth	0.97438	-0.22492	0.7527	0.000999
Temperature	-0.67420	-0.73855	0.2839	0.002997
Chlorophyll a	-0.91184	0.41055	0.1008	0.079920
Salinity	0.99312	-0.11714	0.7100	0.000999
Longitude	-0.75817	-0.65205	0.0015	0.964036
Latitude	-0.60613	0.79537	0.0483	0.316683
Week	-0.19239	-0.98132	0.1388	0.028971

P values based on 1000 permutations.

**Fig 7:**
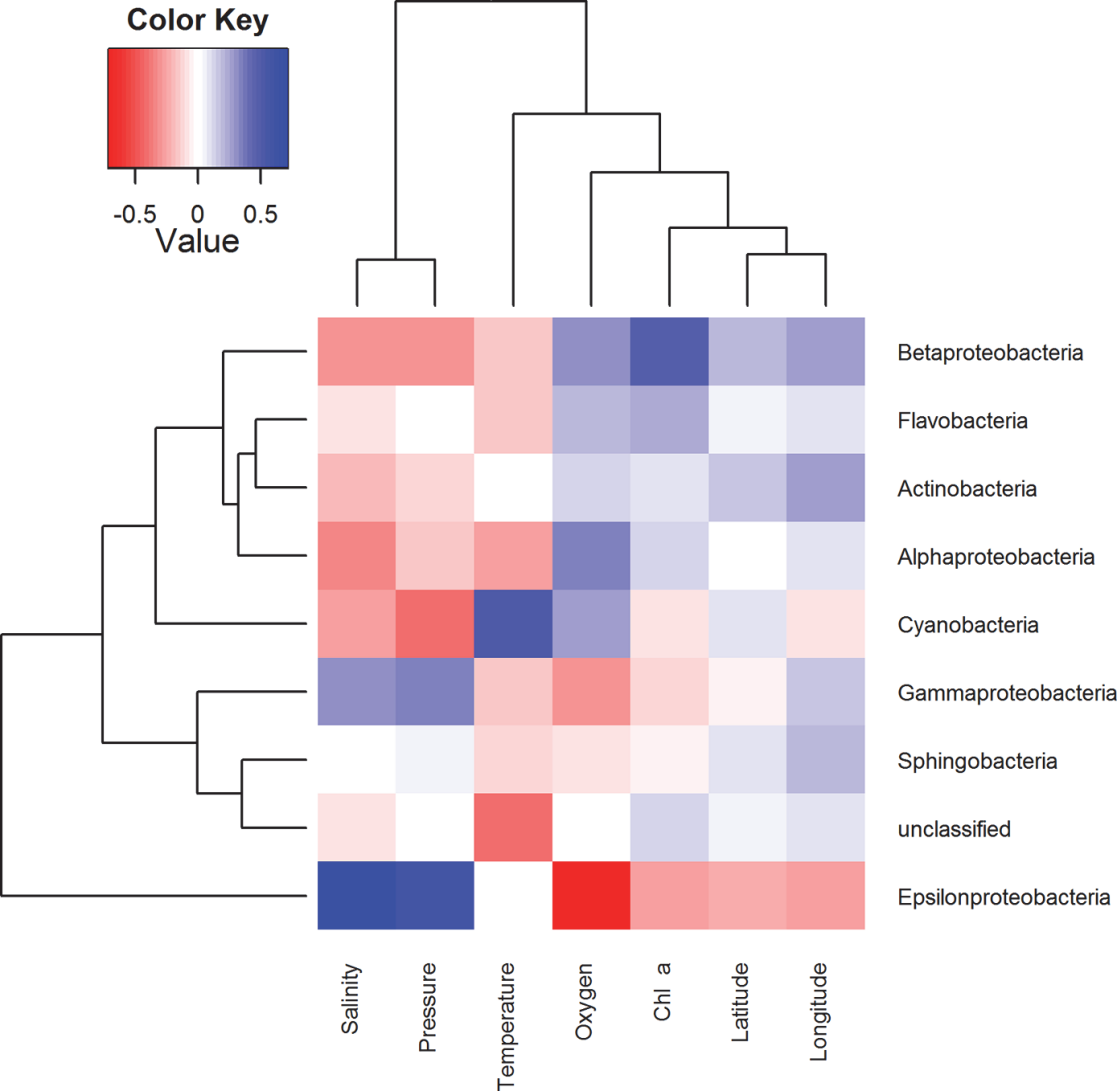
Pearson correlations between environmental parameters (columns) and classes of bacteria (rows). Colors indicate r-values. The dendrograms represent complete linkage clustering of the samples based on the similarities in r-values.

**Fig 8:**
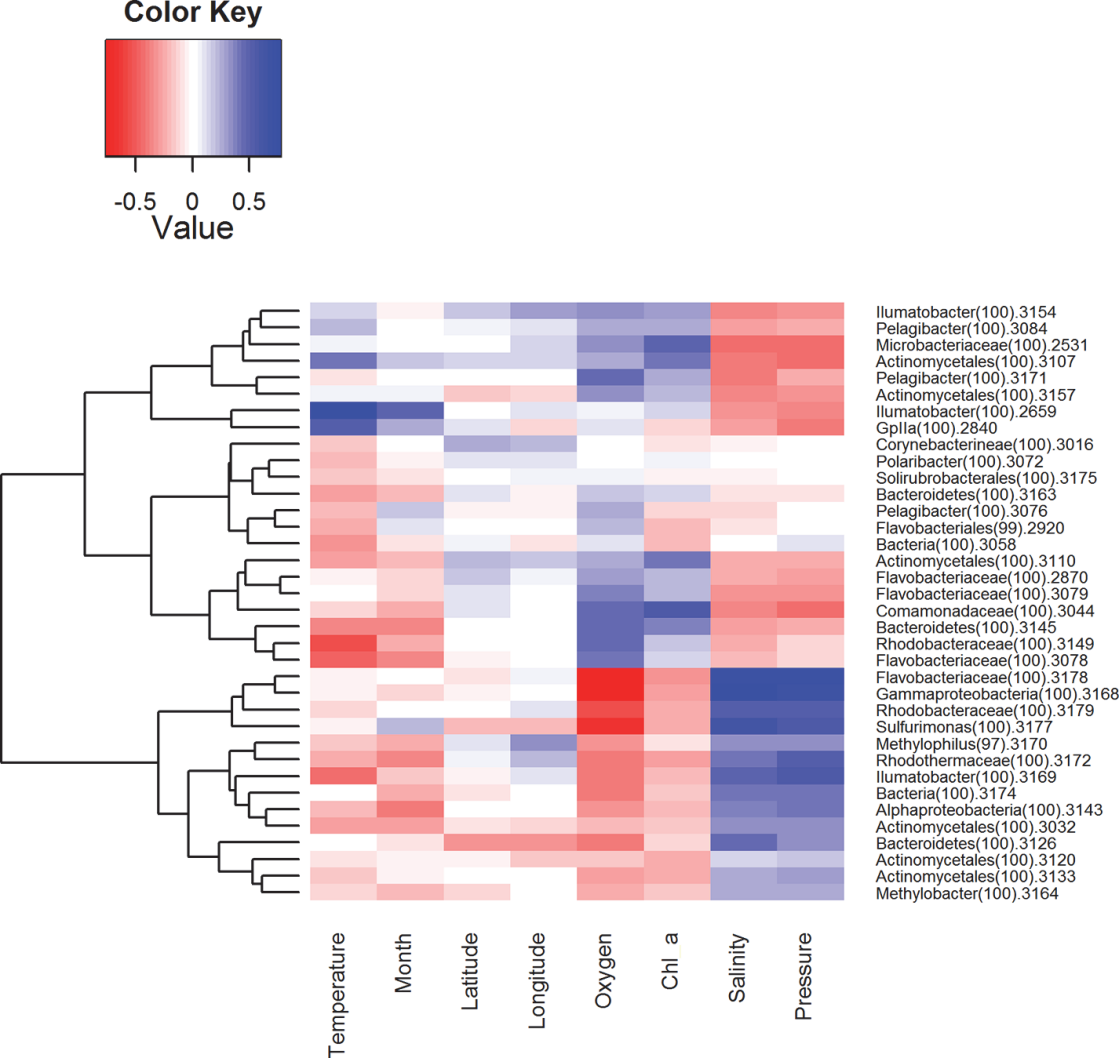
Correlations between environmental parameters and OTUs (>300 sequences in whole dataset). Colors indicate the r-values of Pearson correlations. The dendrograms represent complete linkage clustering of the samples based on the similarities in r-values.

**Fig 9:**
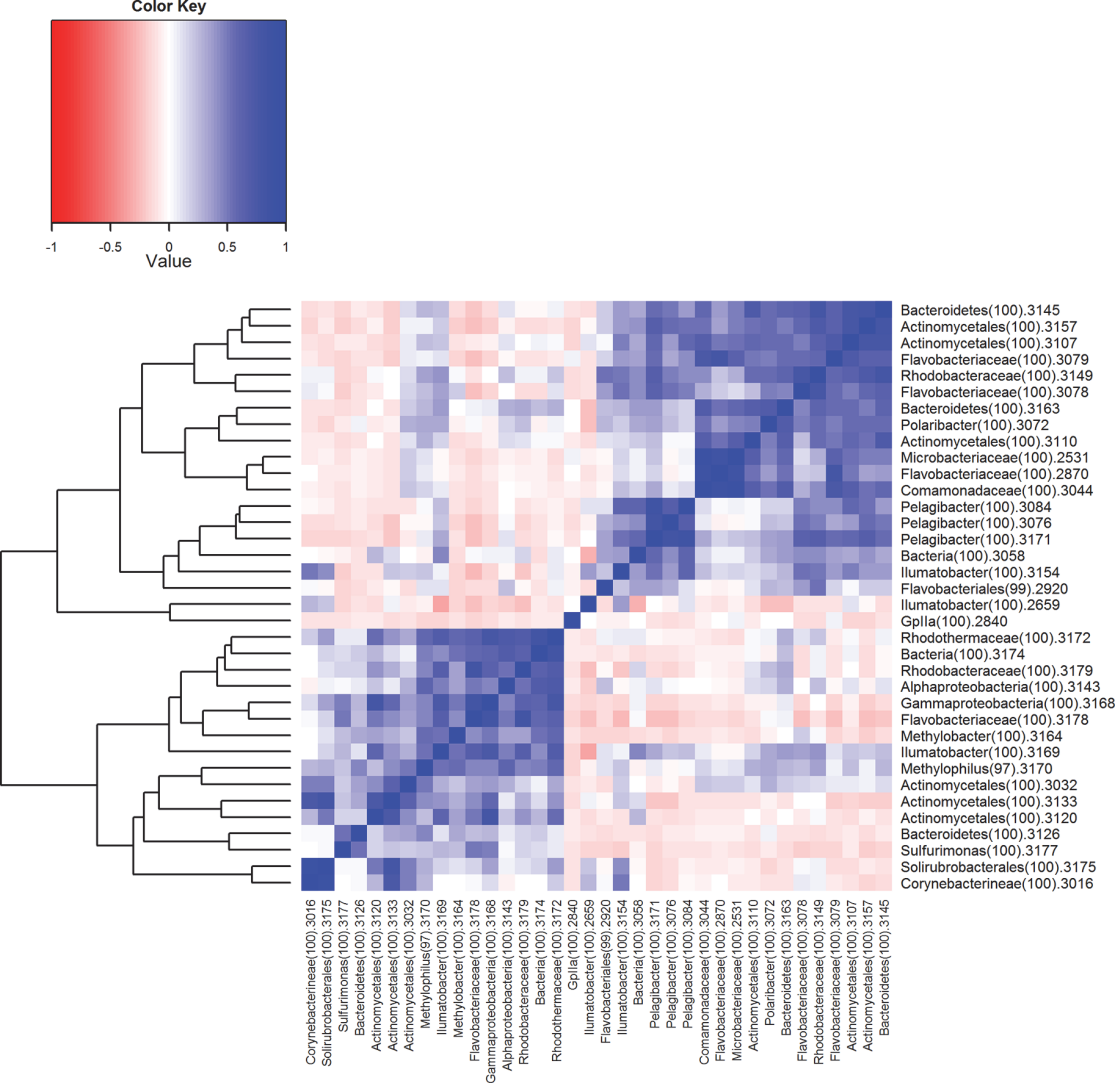
Co-localization analysis between the most abundant ribotypes, which are accompanied by classifications. Colors indicate the r-values of Pearson correlations. The dendrogram represents complete linkage clustering of the samples based on the similarities in r-values.

To examine how the BCC was related to environmental factors, each measured environmental parameter was correlated to the relative abundance of the dominant bacterial classes (Fig 7) of OTUs (Fig 8). These heatmaps illustrate how occurrence patterns of classes differ in relation to environmental conditions and that on OTU level there are subdivisions within these groups. Interactions between microbes were as important as other environmental factors. Therefore, similar correlation analyses were carried out between dominant ribotypes (Fig 9). The clustering achieved in this way is slightly different and provides additional information. The efficiency of this approach is demonstrated by the fact that OTUs with related database matches (isolation source, etc.) clustered together. Therefore, relatively abundant ribotypes with the closest affiliations are given in the same order in Table 4.

## Discussion

### Connection between locally established bacterioplankton community and global redox-specific metacommunity

We used 16S rRNA gene-based community profiling to identify spatiotemporal patterns of bacterial picoplankton (including 0.22 to 5 μm fraction) in the Gulf of Finland. As discussed in detail below, the collected data demonstrate that the dynamics of the BCC in the Gulf of Finland exhibits striking parallels with other OMZ that are also characterized by salinity gradients and a temperate climate, like Chesapeake Bay and the Saanich Inlet [25,58]. This suggests that, in addition to the dynamics of different electron acceptors and donors, salinity also plays an important role.

In community ecology, there is an increasingly popular concept of metacommunities, defined as a set of local communities that are linked by the dispersal of multiple interacting species [59]. Considering the interconnectedness of aquatic ecosystems, we propose that bacterioplankton communities in the OMZ can be considered as a globally distributed redox-specialized metacommunity. In such a framework, OMZs occurring in freshwater and marine water mixing zones should form a salinity-dependent subsystem of this metacommunity. Due high selectivity by salinity- and redox-driven niche partitioning in these systems, species-sorting becomes the driving force behind community assembly in these systems.

The overall phylogenetic makeup of the bacterioplankton community observed on the bacterial class level resembled those routinely found in Baltic Sea pelagic waters using culture independent methods, although an underrepresented fraction of *Gammaproteobacteria* and *Verrucomicrobia* was noted [34,39,42,44,60].

Moreover, many relatively abundant OTUs are closely affiliated with sequences previously isolated from the Baltic Sea (Table 4), as expected due to the long residence time of the Baltic Sea and annually reoccurring patterns of bacterioplankton succession [44]. Two OTUs that managed in some cases to contribute to over 50% of the BCC (Fig 6) can be considered permanent and well-adapted local populations. One of these OTUs, BSNS2840, had an identical match to a sequence isolated from laminae of the Gulf of Finland sediments dating back to the Late Litorina Sea [61]. Hence, this population may have been present in the area for over 3000 years. It was classified as a member of the cyanobacterial clade GpIIa (*Synechococcus* by database affiliation) and it contributed up to 88.7% of the BCC in the surface layer in June, making it the most abundant picocyanobacterium of the dataset (Fig 6). OTU BSNS2840 was also numerous in the winter picoplankton community in the same sampling area [42].

The prevalence of *Cyanobacteria* during summer is typical of the seasonal succession of phytoplankton in the area and is caused by multiple environmental conditions, most notably the increase in temperature and the depletion of nitrates in the surface layer after the spring bloom (Table 2), which gives a distinct advantage to diazotrophic cyanobacteria [62]. The variability of the fraction contributed by *Cyanobacteria* within samples that were collected during same cruise indicates patchy nature of the bloom. In addition, it is important to point out that *Synechococcus* has been shown to occupy the anoxic and dark zone in the central Baltic Sea [38,63] and Chesapeake Bay [25]. The underlying mechanisms behind this phenomenon remain unexplored to our knowledge. The most abundant OTU in the hypoxic/suboxic layer, BSNS3177, was classified as *Sulfurimonas* (genus of *Epsilonproteobacteria*). One characteristic feature of chemolithoautotrophic bacteria in the Baltic Sea is that the majority of cells belong to the Sulfurimonas GD17 group [64], which oxidizes H_2_S with NO^3-^. The prevalence of single domant strain of epsilonproteobacterium is also reflected in our results. Ribotype BSNS3177 contributed to a large fraction of the BCC in the hypoxic/suboxic near-bottom layer at the westernmost stations and at AP2 and AP5 in mid-summer (Fig 6). This suggests dispersion from the Baltic Proper to the Gulf of Finland, as proposed by our previous study [42]. Moreover, the *Sulfurimonas* strain co-occurred with OTU BSNS3126 (Fig 9), classified as *Bacteroidetes*, and both were affiliated with sequences isolated earlier from the anoxic zone of central Baltic Sea (Table 4) [65]. Linkage to the same isolation source was also demonstrated by the co-occurring pair of ribotypes BSNS3168 and BSNS3178, classified as representatives of *Gammaproteobacteria* (affiliated with the SUP05 clade) and *Flavobacteriaceae*, respectively. These correlations probably represent cooperation activities.

The *Epsilon-* and *Gammaproteobacteria* have been identified as major chemolithoautotrophic groups in the central Baltic Sea [40,41,64–67] and also globally in other marine OMZs [31,68]. The GD17 group is capable of a chemotactic response to nitrate [69]. The chemotactic swimming strategies of marine bacteria are especially significant in patchy nutrient seascapes [70], and in OMZs, the corresponding genes have been shown to be over-represented [39,71].

Heterotrophic *Alphaproteobacteria* dominated in the upper oxygenated layer of water. In addition, *Candidatus* Pelagibacter (ribotypes BSNS3076, BSNS3084 and BSNS3171 accounted for 15.9%) was the most abundant genus in the whole dataset. *Candidatus* Pelagibacter belongs to the SAR11 clade, which is the most abundant type of organism in the world’s oceans [12,72,73] and has been found to be numerous in the Baltic Sea as well [35,42]. Another prevalent alphaproteobacterial OTU in oxygenated waters, BSNS3149, was classified as *Rhodobacteraceae* (order *Rhodobacterales*). It had an identical match with a sequence isolated from a coastal North Sea diatom bloom [74]. Both groups, *Rhodobacterales* and SAR11, contribute to the conversion of dimethylsulfoniopropionate to dimethylsulfide [75]. Interestingly, both groups have been found to be numerous in OMZs in both oxic and anoxic layers, but their metabolic adaptations for anaerobic growth remain to be uncharacterized [31].

### Importance of methane and trace metal gradients

Methane is mainly generated in the sediment from which it transfers into the water column. A methane gradient with decreasing concentrations toward the water surface is usually observed in the Baltic Sea [76]. Microbial oxidation of methane in the water column represents an important sink of methane. Different electron acceptors can be used for methane oxidation (sulfate, nitrate, nitrite, iron and manganese), but using oxygen produces a considerably larger energy yield [77].

As a consequence, the hotspot for microbial methane oxidation activity in central Baltic Sea is at the upper part of the redoxcline [78]. Interestingly, the corresponding group consists of only type I methanotrophic bacteria [79,80]. Our results suggest that the same group is also represented in the Gulf of Finland with gammaproteobacterial OTU BSNS3164 (*Methylobacter*) as the most abundant representative. It is important to consider that our samples were collected above and under the redoxcline. In addition, OTU BSNS3143 corresponded via databases to *Methylosinus trichosporium*, a type II methane-oxidizing alphaproteobacterium previously found in surface sediments of the Gotland Deep [81]. Considerably more relevant was betaproteobacterial OTU BSNS3170, classified as *Methylophilus*, an obligate methylotroph common to the Baltic Sea [38,42,82]. All these three groups were more abundant at the 40 m depth (Fig 6).

Redox-sensitive trace metals (e.g. iron and manganese) provide another energy source for microbes and can serve as electron donors above the redoxcline. When oxygenated, insoluble compounds form aggregates that will sink below the redoxcline and can be used as electron acceptors [83–85]. Our results suggest that members of *Actinomycetales* (order of *Actinobacteria*) may inhabit this niche, because several relatively abundant OTUs (BSNS2659, BSNS3154 and BSNS3169) were classified as *Ilumatobacter*. All known and described members of *Acidimicrobiaceae* are capable of iron oxidation [86]. The found OTUs exhibited different patterns of distribution indicating separate strategies. For example, OTU BSNS2659 was more abundant at 5 m in July. However, two other *Ilumatobacteria* and three unclassified *Actinobacteria* (OTUs BSNS3032, BSNS3120, BSNS3133) occurred mainly at 40 m and in the near-bottom layers, supporting the iron-oxidation hypothesis. Interestingly, almost all of these OTUs (except OTU BSNS3169) had nearly identical matches to brackish and temporarily anoxic estuaries (Table 4).

### Spring bloom associated community influenced by freshwater clades

The Gulf of Finland has a relatively large input of freshwater compared to the Baltic Proper and southern basins. This impacts the BCC through the salinity distribution of the gulf and the influx of populations originating from freshwater sources [33–35,44]. Consequently, some relatively abundant OTUs were highly similar to sequences obtained from rivers and lakes (Table 4). Overall, the prevalence of phylogenetic groups considered to be characteristic of freshwater ecosystems, like *Actinobacteria* and *Betaproteobacteria*, exhibited a positive correlation with longitude (Fig 7), i.e. increasing in abundance towards less saline parts of the gulf. This concurs with previous reports of BCC dynamics along the salinity gradient of the Baltic Sea [35].

Annually recurring phytoplankton spring blooms in the Gulf of Finland are co-dominated by diatoms and dinoflagellates and exhibit high spatiotemporal variability [87]. The alternating dominance of bottom-up and top-down interactions result in a succession of heterotrophic bacterial groups with different growth strategies throughout the phytoplankton bloom [74,88]. Although our sampling period covered only part of the spring bloom, groups representing different strategies could be distinguished.

Bacterial lineages considered fast growing “opportunistic” types that utilize dissolved organic matter (DOM) like *Betaproteobacteria* and *Flavobacteria* [88–90] were accompanied by more slow-growing and grazing-resistant members of the AcI lineage of *Actinobacteria* [88,91,92]. Most of the OTUs connected to the spring bloom were clustered together by the correlation analysis (upper 12 OTUs in Fig 7), including the discussed unclassified *Rhodobacteraceae* that were affiliated with the diatom bloom.

Members of *Flavobacteria* are often overrepresented in the spring BCC of the Baltic Sea and are major contributors to the degradation of high molecular weight carbon [39,82,93]. OTUs BSNS2870, BSNS3078 (both classified as *Flavobacteriaceae*) and BSNS3163 (unclassified *Bacteroidetes*) were closely related to sequences previously isolated from the Baltic Sea (Table 4). We identified several OTUs classified as *Polaribacter* (member of *Flavobacteria*), with OTU BSNS3072 as the only relatively abundant representative. This group contributes to the degradation of phytoplankton-derived organic matter via high expression of sulfatases [74].

Overall, *Betaproteobacteria* had the strongest correlation with Chl *a* concentrations, especially unclassified *Comamonadaceae* (BSNS3044) which stood out on the OTU level (Figs 6 and 7, respectively). Members of *Comamonadaceae* have been previously identified in spring bacterioplankton communities on several occasions [39,82]. Moreover, mesocosm experiments have shown a negative impact of higher temperature on this psychrotolerant lineage (Lindh *et al*., 2012).

We identified three relatively abundant members of *Actinobacteria* (OTUs BSNS3107, BSNS3110 and BSNS3157) that were closely related to the freshwater AcI lineage characterized by small cell sizes. The presence of small bacteria can be considered a clear indication of grazing pressure by bacterivorous nanoflagellates. Metagenomic profiling of pelagic and benthic bacteria during the spring (Landsort Deep) reveled that genes for the degradation of polyaromatic hydrocarbons (like cellulose and chitin) belong mainly to *Actinobacteria* and more specifically to *Mycobacterium* [39]. These genes were mainly found in the sediments, and because they are affiliated with aerobic groups, the authors considered sedimentation most likely. In our dataset, OTU BSNS3016, classified as *Corynebacterineae* and closely affiliated with *Mycobacterium*, comprised 8.7% of the total sequences isolated at a depth of 40 m in the beginning of June; therefore, our results support the sedimentation hypothesis.

## Conclusions

The availability of electron acceptors is a critical determinant of the marine ecosystem structure, and we conclude that oxygen concentration is a major environmental factor impacting the BCC in the near-bottom layer of the Gulf of Finland. We conclude that chemolithotrophic groups dispersing from the central Baltic Sea become dominant members of the BCC in the suboxic/anoxic layer when it is formed. Some members of *Actinobacteria* inhabit the layer above the redoxcline and most probably contributing to the oxidation of ferrous iron. Our results also led to the conclusion that the Gulf of Finland has likely a more diverse composition of methanotrophic bacteria than the central Baltic Sea. The BCC at the surface layer was strongly impacted by phytoplankton seasonal succession, as many abundant OTUs in April and May could be associated with the spring phytoplankton bloom. In addition, in the beginning of summer, inorganic nutrient depletion and rising temperatures led to the proliferation of picocyanobacteria. We determined one dominant lineage of *Synechococcus*, which due affiliation contributes to the conclusion that some well-established phylogenetic lineages have persisted in the area for over 3000 years. Furthermore, our results support the emerging pattern of related microbes occupying the OMZs throughout the world and suggests that core of the bacterioplankton community of the Gulf of Finland is part of that a redox-specialized bacterial network, which by definition can be considered a metacommunity.

## Supporting Information

S1 Fig Rarefaction curves outlined from each sample representing the relation between the number of sequences and the number of operational taxonomic units (OTUs) identified with 97% similarity threshold. Different depths are marked with letters: 5 m (a), 40 m (b) and near-bottom layer (c).
(PNG)
