# Supplementary figures and images for: Redox-Specialized Bacterioplankton Metacommunity in a Temperate Estuary
Source: PLoS One. 2015 Apr 10;10(4):e0122304. doi: 10.1371/journal.pone.0122304 (PMC4393233; doi:10.1371/journal.pone.0122304)

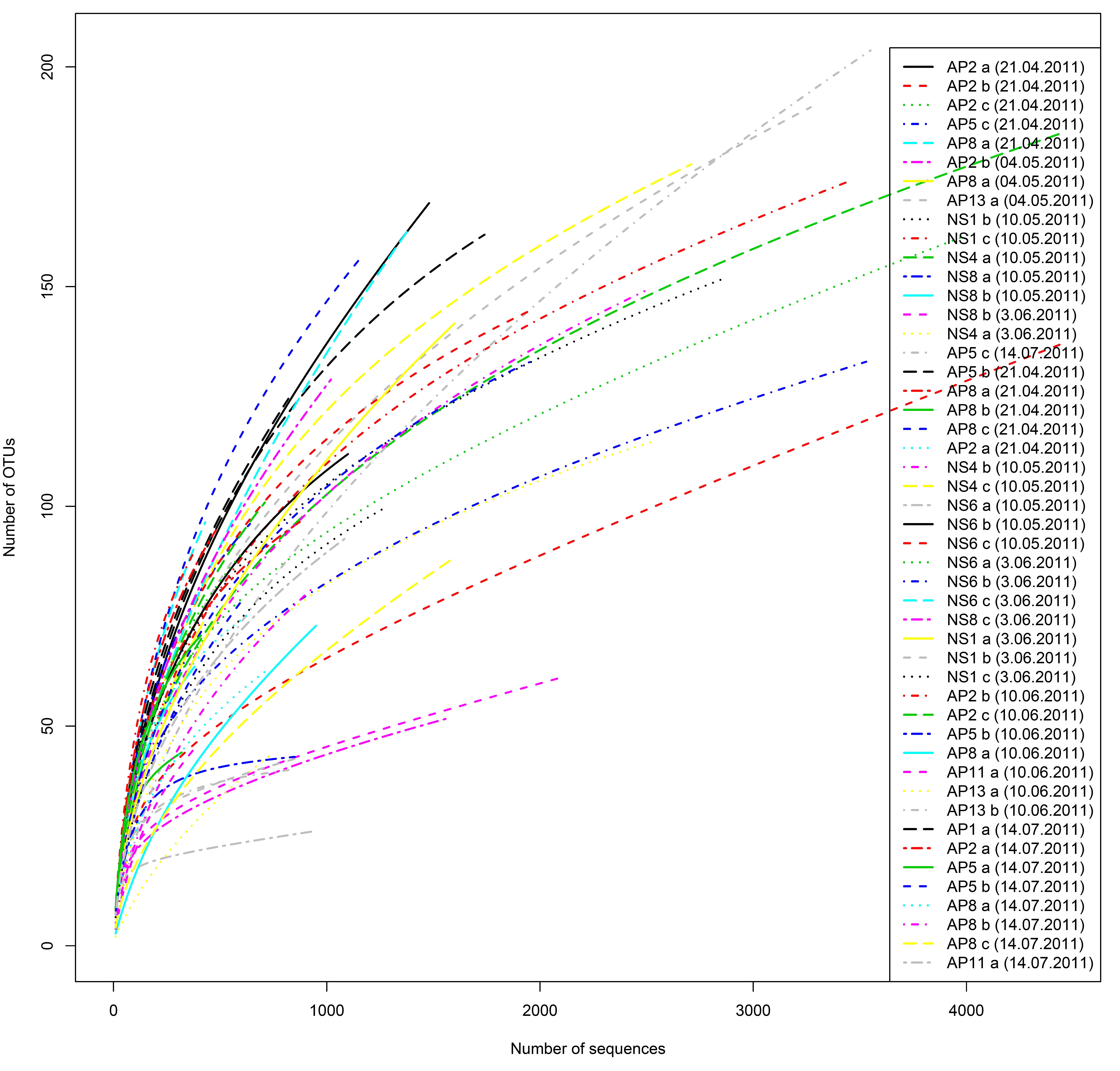

Supplement: S1 Fig — Different depths are marked with letters: 5 m (a), 40 m (b) and near-bottom layer (c). (PNG) [file pone.0122304.s001.png]
